# Abbreviated Multiparametric MR Solution (the “Liver Triple Screen”), the Future of Non-Invasive MR Quantification of Liver Fat, Iron, and Fibrosis

**DOI:** 10.3390/diagnostics14212373

**Published:** 2024-10-24

**Authors:** Gavin Low, Ryan K. W. Chee, Yu Jun Wong, Puneeta Tandon, Florin Manolea, Stephanie Locas, Craig Ferguson, Wendy Tu, Mitchell P. Wilson

**Affiliations:** 1 Department of Radiology and Diagnostic Imaging, University of Alberta, Edmonton, AB T6G 1C9, Canadafmanolea@ualberta.ca (F.M.); locas@ualberta.ca (S.L.); cwfergus@ualberta.ca (C.F.); wtu3@ualberta.ca (W.T.); mitch.wilson@ualberta.ca (M.P.W.); 2 Division of Gastroenterology (Liver Unit), University of Alberta, Edmonton, AB T6G 1C9, Canada; wongyujun1985@gmail.com (Y.J.W.); ptandon@ualberta.ca (P.T.)

**Keywords:** magnetic resonance imaging, magnetic resonance elastography, proton density fat fraction, relaxometry, liver fibrosis, liver fat and iron quantification, chronic liver disease, metabolic dysfunction-associated steatotic liver disease (MASLD), iron overload

## Abstract

**Background/Objectives:** To review the findings of a multiparametric MRI (the “liver triple screen”) solution for the non-invasive assessment of liver fat, iron, and fibrosis in patients with chronic liver disease (CLD). **Methods:** A retrospective evaluation of all consecutive triple screen MRI cases was performed at our institution over the last 32 months. Relevant clinical, laboratory, and radiologic data were analyzed using descriptive statistics. **Results:** There were 268 patients, including 162 (60.4%) males and 106 (39.6%) females. The mean age was 54 ± 15.2 years (range 16 to 71 years). The most common cause of CLD was metabolic dysfunction-associated steatotic liver disease (MASLD) at 45.5%. The most common referring physician group was Gastroenterology at 62.7%. In 23.9% of cases, the reason for ordering the MRI was a pre-existing failed or unreliable US elastography. There were 17 cases (6.3%) of MRI technical failure. Our analysis revealed liver fibrosis in 66% of patients, steatosis in 68.3%, and iron overload in 22.1%. Combined fibrosis and steatosis were seen in 28.7%, steatosis and iron overload in 16.8%, fibrosis and iron overload in 6%, and combined fibrosis, steatosis, and iron overload in 4.1%. A positive MEFIB index, a predictor of liver-related outcomes, was found in 57 (27.5%) of 207 patients. Incidental findings were found in 14.9% of all MRIs. **Conclusions:** The liver triple screen MRI is an effective tool for evaluating liver fat, iron, and fibrosis in patients with CLD. It provides essential clinical information and can help identify MASLD patients at risk for liver-related outcomes.

## 1. Introduction

Chronic liver disease (CLD) poses a significant global health challenge, resulting in approximately 2.1 million deaths worldwide in 2017, marking an increase of 11.4% since 2012 [1,2,3,4]. This underscores the real-world need for effective solutions to address this problem and enhance healthcare outcomes. Liver-related complications, such as portal hypertension and hepatocellular carcinoma, occur most commonly in patients with advanced fibrosis [2]. As fibrosis may be reversible if detected in its early stages, there exists a window during which therapeutic intervention is most effective in improving patient prognosis. Non-alcoholic fatty liver disease (NAFLD), now known as metabolic dysfunction-associated steatotic liver disease (MASLD), is recognized as the leading cause of CLD at 60%, and its prevalence is on the rise [1,3,5,6,7,8]. Currently, it is estimated that MASLD may impact up to 1 billion people globally [6,7,8,9,10]. Additionally, iron overload can serve as both a cause and a consequence of CLD [11,12,13,14,15]. When occurring in MASLD, iron overload can exacerbate the baseline liver dysfunction [11,12,13,14,15]. Therefore, fat, iron, and fibrosis may co-exist as important components in the complex pathophysiology of CLD.

The last decade has seen a rapid growth in technological innovations in medical imaging, leading to improved capabilities in detecting, quantifying, and grading parenchymal liver disease burden. Cutting-edge multiparametric MRI techniques have emerged as reliable surrogate biomarkers for liver fibrosis (e.g., liver stiffness on MR elastography, MRE), liver fat (e.g., MRI-based proton density fat fraction, MRI-PDFF, and magnetic resonance spectroscopy-based proton density fat fraction, MRS-PDFF), and liver iron (R2*). Each of these imaging biomarkers has been validated against histopathology and has received Food and Drug Administration (FDA) approval for clinical use [16,17,18,19,20,21,22,23,24,25,26,27,28]. Compared to liver biopsy, these MRI methods are non-invasive and can sample a large volume of the liver, making them more attractive to patients and clinicians for longitudinal disease monitoring and the assessment of treatment response. Emerging evidence indicates that imaging biomarkers such as liver stiffness can be combined with serum biomarkers such as Fibrosis-4 (FIB-4) to stratify the risk of liver-related events in patients with MASLD [29,30]. These events include the development of ascites, varices requiring intervention, encephalopathy, hepatocellular carcinoma, and death.

Similar to MRI, a variety of quantitative ultrasound (US) techniques are available clinically for evaluating liver stiffness, the two main methods in mainstream use include transient elastography and shear wave elastography. More recently, several proprietary US solutions have also been developed for liver fat quantification, such as controlled attenuation parameter (Echosens), attenuation imaging (Canon), attenuation coefficient (Fujifilm), US-guided attenuation parameter (General Electric), and US-derived fat fraction (Siemens) [31]. Unlike MRI, US-based methods are more cost-effective and widely available, making them a more practical first-line imaging tool for evaluating patients with CLD. The American Association for the Study of Liver Diseases (AASLD) practice guidelines recommend that “***either US-based elastography or MRE can be used to stage liver fibrosis in patients with CLD and that depending on local availability and expertise, it would be reasonable to perform MRE when concomitant cross-sectional imaging is needed or for patients where US-based methods might be compromised***” [2].

Over the last 2–3 years, our institution has performed an abbreviated multiparametric MRI protocol, known as the “***Liver Triple Screen***” to simultaneously detect and quantify liver fat, iron, and fibrosis in patients with CLD. This examination integrates a minimal set of MRI sequences to reduce scanning time and improve patient throughput. Our institution stands among a select number of centers globally that offer this examination as part of routine clinical care. Given that the current gold standard liver biopsy is imperfect due to sampling error and suboptimal concordance with fibrosis evaluation, multiparametric MRI has emerged as a potential solution to this unmet clinical need [32,33,34,35,36,37,38]. Herein, the objective of this study was to conduct a comprehensive retrospective analysis of our first 268 consecutive patients who underwent the liver triple screen MRI and to report on this experience. The study aimed to gather data to obtain a better understanding of our patient cohort, referring physician base, the reasons for ordering a triple screen MRI, the technical examination success rate, causes for technical examination failure, the prevalence of liver fibrosis, steatosis or iron overload (or any combination of these), the identification of patients at potential risk of liver-related events, as well as the prevalence of incidental findings on the triple screen MRI, and their potential significance.

## 2. Materials and Methods

A retrospective observational study was conducted at a single Canadian institution. This facility serves as a tertiary referral center for CLD and covers a catchment area of roughly half the province of Alberta. This study was conducted in accordance with the Declaration of Helsinki and the protocol was approved by the Ethics Committee of the University of Alberta (Pro00141219) on 3 April 2024. The study received a waiver of written patient consent, as all cases were anonymized, and personal identifying information was removed. This study included all consecutive outpatients who underwent a triple screen MRI from 1 July 2021 to 29 February 2024 (32 months).

### 2.1. Triple Screen MRI Protocol

The triple screen MRI protocol uses an 18-channel body matrix array coil on a 1.5-T MRI system (Magnetom Aera, Siemens Healthcare, Erlangen, Germany) that features a wide bore that is suitable for larger patients and those with claustrophobia. This abbreviated protocol incorporates a minimal set of MRI sequences to reduce scanning time to approximately 15 to 20 min. The examination does not require the use of intravenous gadolinium contrast. The examination’s MRE component is conducted first, followed by fat and iron quantification, which are acquired simultaneously.

**Figure 1:**
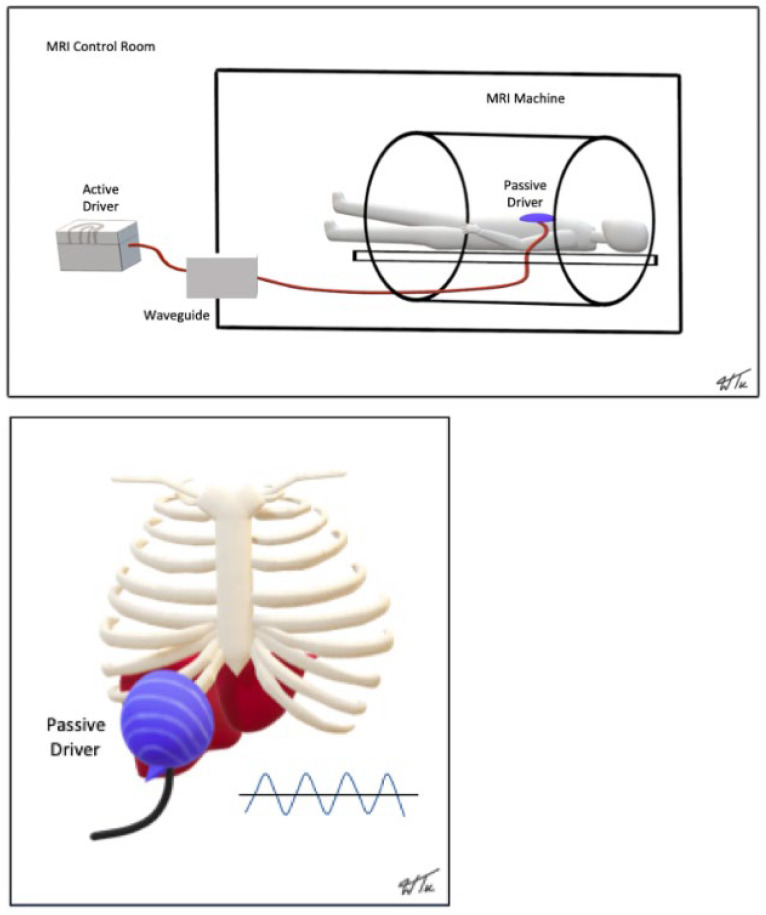
Schematic diagram showing the external setup for MRE, including patient positioning and placement of the soft driver over the right upper abdomen. *Reproduced with permission (Springer Nature and Copyright Clearance Center) from “Multiparametric MR assessment of liver fat, iron, and fibrosis: a concise overview of the liver Triple Screen”. Low G et al., Abdominal Radiology (New York) 2023;48(6):2060–2073* [11].

**MRE:** The physical setup for MRE involves an external driver system that generates acoustic waves at a vibration frequency of 60 Hz. An 18 cm soft driver is securely fastened to the right upper abdomen/rib cage using an abdominal band while the patient lies supine on the MRI table. The external driver is connected to the soft driver via a hollow plastic tube and produces mechanical waves that pass through the body at various speeds based on inherent tissue stiffness (Figure 1). Subsequently, four 10-mm non-contiguous axial sections of the liver, typically at its widest parts, are obtained using a modified phase contrast sequence, either a 2D-gradient echo (GRE) sequence or a 2D-spin echo echoplanar (SE-EPI) imaging sequence. The 2D-GRE sequence was utilized for all MRE examinations performed between 1 July 2021 and 30 June 2023. As part of a service upgrade, the GRE sequence was later replaced with the 2D SE-EPI sequence for all MRE examinations from 1 July 2023 onwards. The technical parameters for both MRI sequences are detailed in Table 1. Each MRE acquisition requires an end-expiration breath-hold lasting 16 to 17 s. This process creates elastograms consisting of grayscale and color maps and a 95% confidence map overlaid with crosshatching. Liver stiffness measurements are obtained from the elastograms by delineating regions of interest (ROI) in the right lobe of the liver while avoiding the liver borders, fissures, gallbladder fossa, large vessels, and crosshatched areas. The grading of liver stiffness is based on the following recommended published reference ranges [39,40,41,42]:

➢ *normal: <2.5 kPa;*
➢ *normal or inflammation: 2.5 to 2.9 kPa;*
➢ *stage 1 to 2 fibrosis: 2.9 to 3.5 kPa;*
➢ *stage 2 to 3 fibrosis: 3.5 to 4 kPa;*
➢ *stage 3 to 4 fibrosis: 4 to 5 kPa;*
➢ *stage 4 fibrosis/cirrhosis: >5 kPa.*

**Table 1:** The technical parameters for the 2D GRE (gradient echo) sequence and the 2D SE-EPI (spin echo echoplanar imaging) sequence used for MRE.

	GRE	SE-EPI
**TR**	25 ms	1200 ms
**TE**	21.5 ms	47 ms
**Slice thickness**	10 mm	10 mm
**Flip angle**	12°	90°
**Averages**	1	1
**FOV**	420 mm	380 mm
**Matrix**	100 × 100%	128 × 100%
**Bandwidth**	399 Hz/pixel	2170 Hz/pixel
**Acceleration factor**	2	2

*TR—time to repetition, TE—time to echo, FOV—field of view.*

**Fat and Iron Quantification:** A confounder-corrected chemical-shift encoded multi-echo Dixon sequence (LiverLab, Siemens Healthcare, Erlangen, Germany) is used to perform non-invasive fat and iron quantification. This involves an axial six-echo 3D spoiled gradient echo sequence with an 18–20 s breath-hold acquisition (Table 2). The sequence automatically generates the following data on PACS (picture archiving and communication system):

- A report of the mean MRI-PDFF (%) and mean R2* (s^−1^) for the entire liver (segmentectomy);
- A report of the mean MRI-PDFF (%) and mean R2* (s^−1^) for a selected ROI placed in the right hepatic lobe.

**Table 2:** The technical parameters for the multi-echo Dixon sequence and MRS (magnetic resonance spectroscopy).

	Multi-Echo Dixon	MRS
**TR**	15.6 ms	3000 ms
**TE**	2.38, 4.76, 7.14, 9.52, 11.9, 14.28 ms	12, 24, 36, 48, 72 ms
**Slice thickness**	10 mm	n/a
**Flip angle**	4°	90°
**Averages**	1	1
**FOV**	380 mm	n/a
**Matrix**	160 × 79%	n/a
**Bandwidth**	1080 Hz/pixel	n/a
**Acceleration factor**	2	n/a

*TR—time to repetition, TE—time to echo, FOV—field of view,* *n/a—not applicable.*

Following this, MRS is performed on a 27 cm^3^ voxel placed in the right hepatic lobe. MRS is a multi-echo STEAM (stimulated acquisition mode) sequence with high-speed T2 correction and involves a 15 s breath-hold acquisition (Table 2). An automatic spectroscopy report of the mean MRS-PDFF (%) of the 27 cm^3^ voxel is generated on PACS. For both the multi-echo Dixon ROI and the MRS voxel, the MRI technologist selects the sampling location in the right lobe and confirms its placement with the radiologist while the patient is being scanned.

The grading of liver fat is based on the following published recommended reference ranges [9]:

➢ *Grade 1, mild steatosis: PDFF 5–14%;*
➢ *Grade 2, moderate steatosis: PDFF 15 to 24%;*
➢ *Grade 3, severe steatosis: PDFF ≥ 25%*.

For liver iron, R2* is converted to a LIC (liver iron concentration) using the formula: LIC = R2* × 0.032 mg/g on a **1.5-T** MRI system or LIC = R2* × 0.017 mg/g on a **3-T** MRI system. The grading of liver iron is based on the following published recommended reference ranges [43]:

➢ *Borderline iron overload: LIC of 1.8 to 3.2 mg/g*
➢ *Mild iron overload: LIC of >3.2 to 7 mg/g*
➢ *Moderate iron overload: >7 to 15 mg/g*
➢ *Severe iron overload: >15 mg/g*

### 2.2. Data Analysis and Extraction

All MR images are displayed in Digital Imaging and Communications in Medicine (DICOM) format on a PACS workstation. Relevant clinical, laboratory, and radiologic data were retrieved from PACS and the provincial electronic health record. The extracted data was anonymized and entered into a password-protected Excel spreadsheet for analysis.

### 2.3. AI Tools

AI tools were used only to identify grammatical errors and to spell-check the manuscript.

### 2.4. Statistical Analysis

Categorical variables are reported as values and percentages. Continuous variables are reported as the mean ± standard deviation. Statistical analysis of descriptive statistics was performed on IBM SPSS (version 29).

## 3. Results

### 3.1. Patient Demographics and Etiology of CLD

**Figure 2:**
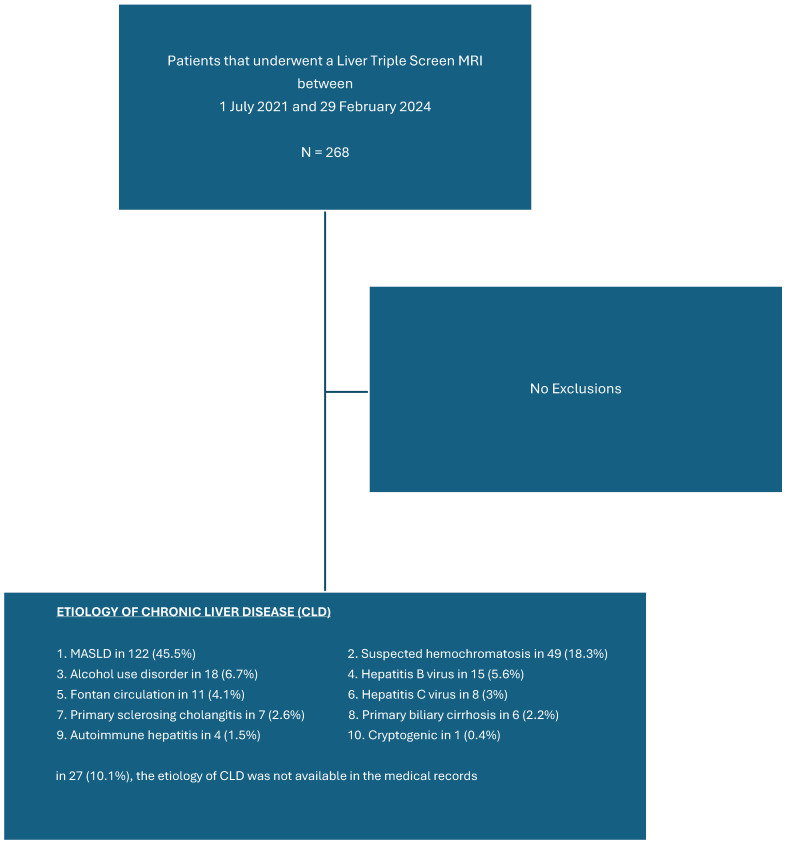
The primary etiologies of CLD in the study population.

This study involved 268 patients, including 162 (60.4%) males and 106 (39.6%) females. The mean age was 54 ± 15.2 years (range 16 to 71 years). The mean weight was 93.8 ± 25.4 kg (range 47.2 to 210 kg). The body mass index (BMI) ranged from 17.2 to 66.3 kg/m^2^, with a mean BMI of 32.2 ± 8.1 kg/m^2^. Subgroup analysis showed that 149 of 268 patients (55.6%) were obese (BMI ≥ 30 kg/m^2^), with the mean BMI in the obese cohort being 37.9 ± 6.1 kg/m^2^, and with 28.9% with a BMI of ≥40 kg/m^2^. The primary etiologies of CLD in the study population are listed in Figure 2. Additionally, 93 patients (34.7%) had hypertension, 68 (25.4%) had diabetes, and 62 (23.1%) had dyslipidemia.

### 3.2. Serum Laboratory Data

Biochemical tests taken within 4 months of the MRI are listed in Table 3 below:

Additionally, serum tests were available to calculate the FIB-4 score in 221 patients. The mean FIB-4 score was 2.8 ± 2.4 (range 0.2 to 18.4). A FIB-4 ≥ 1.3 was found in 74.2%. The AASLD practice guidance on MASLD suggests that a FIB-4 of <1.3 can be used to exclude patients with advanced fibrosis [44].

### 3.3. Referring Physician Groups

The referring physician groups involved in requisitioning the liver triple screen MRI examinations are listed in Table 4 below.

Our patient population with CLD could be broadly classified as follows:

- Patients with a failed or unreliable US elastography: 65 (23.9%);
- Patients with suspected hemochromatosis: 52 (19.4%);
- Patients with a Fontan circulation: 10 (3.7%);
- Others: 142 (53%).

### 3.4. MRI Technical Failures

**Figure 3:**
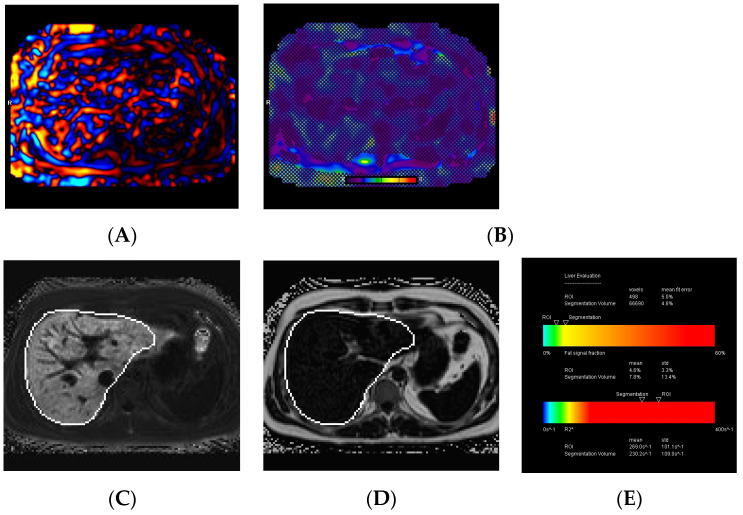
A 23-year-old female with juvenile hereditary hemochromatosis. The images show poor-quality disorganized mechanical waves (**A**), and a non-diagnostic elastogram (**B**), on MRE due to signal degradation from the iron overload. MRI-PDFF map (**C**), R2* map (**D**), and the liver evaluation table (**E**), demonstrate mild steatosis (7.8%) and moderate iron overload (R2* of 230.2 s^−1^ and a LIC of 7.4 mg/g) based on segmentation.

**Figure 4:**
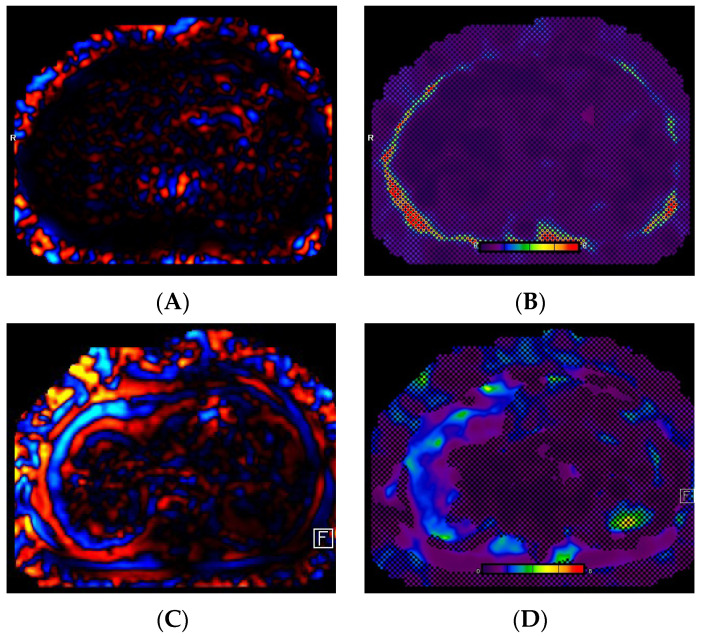
A 53-year-old female. The images show low-amplitude mechanical waves (**A**) and a non-diagnostic elastogram (**B**) on MRE secondary to mechanical driver dysfunction. Repeat imaging following driver repair showed satisfactory wave (**C**) and elastogram (**D**) images and a normal liver stiffness of 1.9 kPa.

Out of the 268 cases, 200 (74.6%) were performed on the 2D SE-EPI MRI sequence, while the remaining 68 (25.4%) used the 2D-GRE MRI sequence. There were 17 cases (6.3%) of technical failure on MRI, with 16 cases (6%) related to MRE failure and 1 case (0.4%) related to iron quantification failure. All technical failures occurred on the 2D-GRE MRI sequence, while all 200 cases using the 2D SE-EPI MRI sequence were satisfactory. The causes of MRE failure included iron overload in 12 cases (75%), mechanical driver dysfunction in 2 cases (12.5%), poor paddle contact in 1 case (6.3%), and an unclear cause of failure in 1 case (6.3%) (Figure 3 and Figure 4). A fat and water swap artifact was responsible for the single case of iron quantification failure.

### 3.5. Liver Triple Screen MRI Findings

Findings on the triple screen MRI include:

- a mean liver stiffness of 3.3 ± 1.8 kPa (range 1.4 to 14.9 kPa);
- a mean PDFF of 11.0 ± 8.7% (range 1.2 to 42.0%);
- a mean liver LIC of 1.9 ± 2.8 mg/g (range 0.1 to 31.3 mg/g).

**Figure 5:**
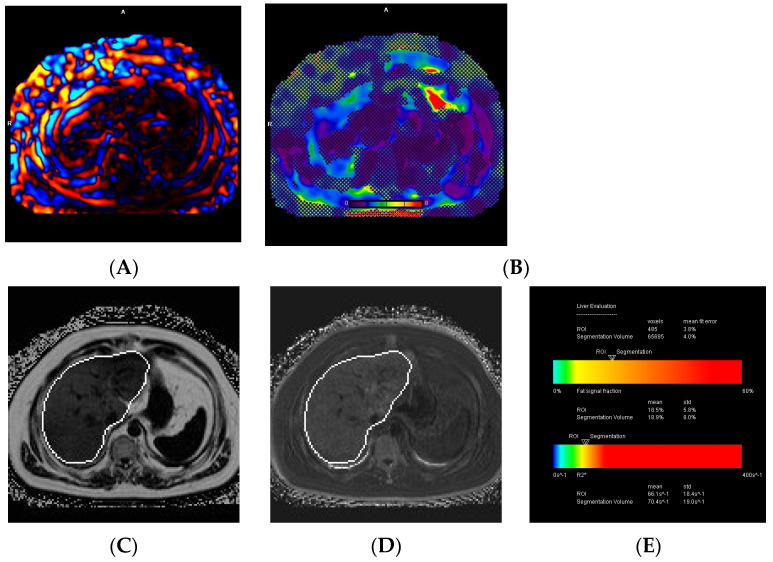
A 60-year-old male with MASLD. Wave (**A**) and elastogram (**B**) MRE images show a normal or inflamed liver with a stiffness of 2.5 kPa. MRI-PDFF map (**C**), R2* map (**D**), and the liver evaluation table (**E**) demonstrate moderate steatosis (18.9%) and borderline iron overload (R2* of 70.4 s^−1^ and a LIC of 2.3 mg/g) based on segmentation.

**Figure 6:**
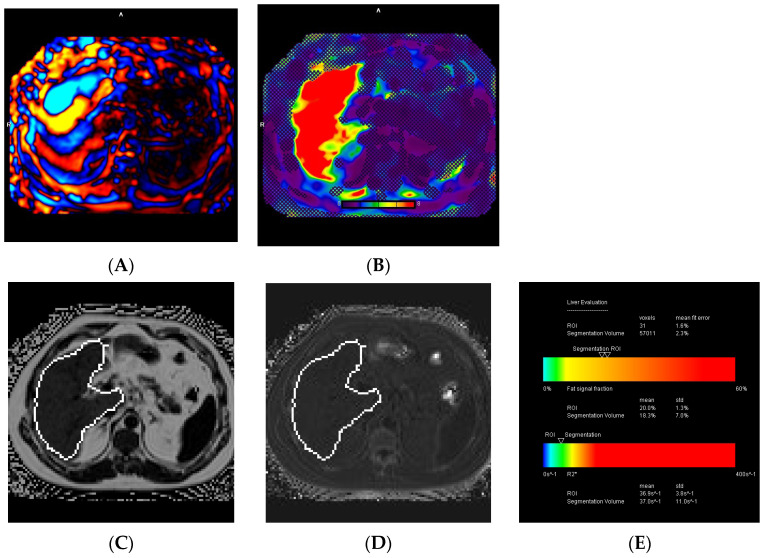
A 53-year-old male with MASLD. Wave (**A**) and elastogram (**B**) MRE images show a cirrhotic liver with a stiffness of 8.0 kPa. MRI-PDFF map (**C**), R2* map (**D**), and the liver evaluation table (**E**) demonstrate moderate steatosis (18.3%) and a normal liver iron (R2* of 37 s^−1^ and a LIC of 1.2 mg/g) based on segmentation.

**Figure 7:**
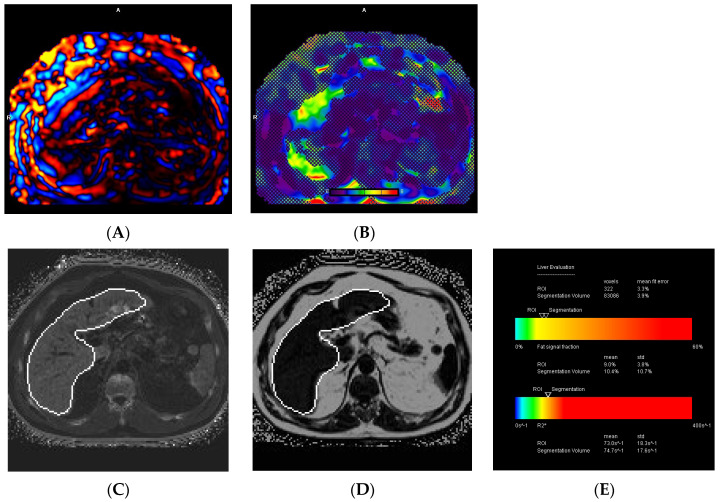
A 63-year-old male with HCV. Wave (**A**) and elastogram (**B**) MRE images show a heterogeneous fibrosis distribution equating to stage 2 to 3 fibrosis with a stiffness of 3.8 kPa. MRI-PDFF map (**C**), R2* map (**D**), and the liver evaluation table (**E**) demonstrate mild steatosis (10.4%) and borderline iron overload (R2* of 74.7 s^−1^ and a LIC of 2.4 mg/g) based on segmentation.

Excluding the 16 patients who experienced technical failure on MRE, normal liver stiffness was observed in 91 (34%) patients, while 44 (16.4%) had normal or inflamed livers, 39 (14.6%) had stage 1 to 2 fibrosis, 26 (9.7%) had stage 2 to 3 fibrosis, 18 (6.7%) had stage 3 to 4 fibrosis, and 34 (12.7%) had cirrhosis. A liver stiffness ≥ 3.3 kPa was found in 95 (37.7%) patients. Excluding the one patient who had a technical failure in liver iron quantification, MRI revealed normal liver iron in 208 (77.9%) patients, borderline iron overload in 35 (13.1%), mild iron overload in 15 (5.6%), moderate iron overload in 7 (2.6%), and severe iron overload in 2 (0.7%). Furthermore, MRI showed normal liver fat in 85 (31.7%) patients, mild steatosis in 124 (46.3%), moderate steatosis in 26 (9.7%), and severe steatosis in 33 (12.3%). Among the total study population of 268 patients, subgroup analysis revealed that 77 (28.7%) had combined liver fibrosis and steatosis, 45 (16.8%) had combined liver iron overload and steatosis, 16 (6%) had combined liver fibrosis and iron overload, and 11 (4.1%) had combined liver fibrosis, steatosis, and iron overload (Figure 5, Figure 6 and Figure 7).

### 3.6. Correlating Serum Tests with MRI

There were 100 MASLD patients that had a FIB-4 and MRE performed for correlation. Using a FIB-4 cut-off of 1.3, we found that FIB-4 had a sensitivity, specificity, positive and negative predictive value of 76.5% (95% CI, 50.1 to 93.2%) 27.7% (95% CI, 18.5% to 38.6%), 17.8% (95% CI, 13.9 to 22.6%) and 85.2% (95% CI, 69.5 to 93.6%) for predicting advanced fibrosis (F3-4) in our cohort using MRE as the reference standard. A FIB-4 < 1.3 was seen in 23.5% of cases (false negative rate) that had advanced fibrosis on MRE. We also explored the proportion of patients at high-risk of developing liver-related events, defined as being MEFIB positive (MRE ≥ 3.3 kPa and FIB-4 ≥ 1.6) [29]. An MRE ≥ 3.3 kPa was seen in 37.7% of 252 patients. A FIB-4 ≥ 1.6 was seen in 62.4% of 221 patients. In total, a positive MEFIB index was observed in 57 (27.5%) of 207 patients. Please see the Discussion section for more details on MEFIB.

There were 151 patients who had a serum ferritin, and MRI-derived LIC was performed for correlation. We found that an elevated serum ferritin of >500 µg/L had a sensitivity, specificity, positive and negative predictive value of 61.9% (95% CI, 45.6 to 76.4%), 89.9% (95% CI, 82.7 to 94.9%), 70.3% (95% CI, 56.3 to 81.3%), and 86.0% (95% CI, 75.1% to 87.9%) for predicting liver iron overload in our cohort using MRI as the reference standard. A normal serum ferritin was found in 38.1% of cases (false negative rate) that had iron overload on MRI.

### 3.7. Incidental Findings

Excluding simple cysts of no clinical consequence, there were 44 incidental findings in 40 (14.9%) of the 268 MRI examinations. These include 14 new findings and 30 known findings, listed below:

- **Liver:** 10 cases of liver hemangiomas (2 of which are new), 1 new case of a regenerating or dysplastic liver nodule, 3 cases of iron-spared liver nodules (2 of which are new), 1 new case of pseudolipoma of Glisson’s capsule, and 1 known case of an intrahepatic portal-systemic shunt;
- **Gallbladder and Biliary:** 11 known cases of uncomplicated cholelithiasis, 1 new case of choledocholithiasis with upstream biliary obstruction, and 1 new case of adenomyomatosis;
- **Genito-urinary:** 1 new case of renal cell carcinoma, 2 known cases of renal angiomyolipoma, and 1 new case of hydronephrosis;
- **Adrenal:** 5 cases of adenomas (2 of which are new) and 2 cases of myelolipomas (1 of which is new);
- **Pancreas:** 1 known case of an intrapancreatic splenic tissue;
- **Gastrointestinal:** 1 new case of a hiatus hernia;
- **Vascular:** 1 known case of an abdominal aortic aneurysm;
- **Thoracic:** 1 known case of a pleural effusion.

## 4. Discussion

This study revealed that MASLD is the most common cause of CLD in our population being referred for the liver triple screen MRI. Our findings agree with the medical literature that has reported MASLD as the most common cause of CLD globally [5,7,45,46,47,48]. This growth in MASLD cases has been attributed to the combination of obesity, diabetes, and metabolic syndrome that has reached epidemic levels in some Western countries [47]. Despite this, MASLD is still likely to be underreported as it remains clinically silent until late [48]. Hemochromatosis contributed to 18.3%, making it our study’s second most common cause of CLD. This prevalence exceeds that of alcohol (6.7%), HBV (5.6%), or HCV (3%), which is in contradistinction to the medical literature, where the latter etiologies are reported as being more common [4,46]. Our institution’s role as a provincial referral center for hemochromatosis can explain this discrepancy, which skews our data. A significant number of these patients are referred for MRI to assess liver iron levels and exclude advanced fibrosis. In total, the majority of our referrals, 62.7%, came from the Gastroenterology and Hepatology Unit, our largest physician partner group. Infectious Diseases made up 4.5% of the referrals, as some of these physicians oversee the care of patients with HBV and HCV. Internal Medicine made up 14.6% and was the main source of referrals for patients with hemochromatosis. Family Medicine was the second largest group at 17.2%. This probably reflects the reality that many patients are managed in the community due to the high prevalence of CLD.

The non-invasive imaging assessment of liver fat and fibrosis in patients with MASLD can be performed on US or MRI. Triple screen MRI offers the additional advantage of simultaneous quantification of liver iron, a capability not available with US-based methods. In a study involving 581 MASLD patients, Beyer et al. compared the diagnostic performance of MRI-PDFF with US-derived controlled attenuation parameter (CAP) for detecting liver steatosis using histology as the reference standard. The study found MRI-PDFF to be superior to CAP for detecting steatosis grades ≥ 2 and ≥3, while both techniques performed similarly for steatosis grades ≥ 1 [49]. Comparing MRI-PDFF to CAP, AUROCs were 1.00 (95% CI, 0.99 to 1.0) vs. 0.95 (95% CI, 0.91 to 0.99) for grades ≥ 1, 0.77 (95% CI, 0.72 to 0.82) vs. 0.6 (95% CI, 0.55 to 0.65) for grades ≥ 2, and 0.81 (95% CI, 0.76 to 0.87) vs. 0.63 (95% CI, 0.56 to 0.70) for grade 3 [49]. A systematic review and pooled analysis of 230 patients with MASLD also compared MRE with transient elastography (TE) and found MRE to have superior accuracy in determining fibrosis grade [18]. The AUROC for MRE was higher than that of TE for all fibrosis stages. These were 0.87 vs. 0.82 (*p* = 0.04) for stages ≥ 1, 0.92 vs. 0.87 for stages ≥ 2 (*p* = 0.03), 0.93 vs. 0.84 for stages ≥ 3 (*p* = 0.001), and 0.94 vs. 0.84 for stage 4 (*p* = 0.005) [18]. In a separate study by Park et al. involving 104 MASLD patients, MRI was found to be more effective than US for detecting liver fibrosis and steatosis using histology as the reference standard [50]. MRE showed an AUROC of 0.82 (95% CI, 0.74 to 0.91) for fibrosis grades ≥ 1, outperforming TE, which had an AUROC of 0.67 (95% CI, 0.56 to 0.78). For steatosis grades ≥ 1, MRI-PDFF demonstrated an AUROC of 0.99 (95% CI, 0.98 to 1.00) compared to 0.85 (95% CI, 0.75–0.96) for CAP [50].

The studies above indicate that MRI is superior to ultrasound (US) in quantifying fat and fibrosis. However, due to cost and accessibility considerations, US is preferred as the primary imaging tool. MRI is better suited for problem-solving, particularly when US is ineffective or when there is a need to measure liver iron. The medical literature suggests that nearly 20% of patients experience failed or unreliable results with US elastography, primarily due to obesity, with other factors being ascites and limited operator experience [51]. In our study, 23.9% of patients underwent triple screen MRI because their pre-existing US elastography failed or was unreliable. It is worth noting that all these patients had successful MRIs, showing that MRI can overcome these challenges. Additionally, no technical issues arose during MRI scans due to high BMI (55.6% of the study population had a BMI of at least 30 kg/m^2^). Out of 268 cases, there were 17 (6.3%) technical failures during triple screen MRI, with all but one occurring during MRE. Iron overload was the main cause of technical failure (n = 12). However, since switching from the GRE sequence to the SE-EPI sequence on our 1.5-T MRI system, which is more resilient to signal degradation from iron overload, there have been no technical failures in all 200 cases scanned. A study by Wagner et al. of 781 MRE examinations found a higher technical failure rate on 3-T MRI (15.3%) compared to 1.5-T MRI (3.5%), with the main causes of failure being iron deposition, high BMI, and massive ascites [52].

Our study found that 66% of the patients had liver fibrosis, 68.3% had steatosis, and 22.1% had iron overload. Cirrhosis was identified in 12.7%, severe steatosis in 12.3%, and severe iron overload in 0.7%. Among the 268 patients, 28.7% showed both fibrosis and steatosis, 16.8% had both steatosis and iron overload, and 6% exhibited both fibrosis and iron overload. Furthermore, 4.1% of the patients demonstrated fibrosis, steatosis, and iron overload concurrently. Our data suggest that combined parenchymal abnormalities are a relatively common finding in CLD. Multiparametric screening for assessing liver fibrosis, fat, and iron could offer a comprehensive imaging profile of the disease burden. This has the potential to improve our understanding of the disease process and may help guide clinical management and treatment options.

Liver biopsy has been considered the “imperfect gold standard” for fibrosis assessment in patients with CLD due to its invasive nature, sampling error, and interobserver variability [32,33,34,35,36,37,38]. Here, the triple-screen MRI offers a non-invasive alternative for fibrosis assessment in CLD, particularly for those with a failed US elastography or who have contraindications to biopsy. Given the asymmetrical fibrosis distribution within the liver, biopsy is subject to sampling error, which may be mitigated by using the triple-screen MRI where volumetric assessment can be performed (Figure 7). In our institution, the estimated cost to undergo a triple-screen MRI was not significantly higher than a liver biopsy (CAD 700 for MRI vs. CAD 835 for biopsy, including post-procedure observation costs). Our study findings also suggest that other non-invasive alternatives to liver biopsy, such as serum markers including FIB-4 and ferritin have suboptimal accuracy on their own for excluding advanced fibrosis and liver iron overload, respectively, when assessed against MRI. The false negative rate was 23.5% for a FIB-4 of < 1.3 and 38.1% for ferritin.

Emerging evidence indicates that non-invasive scoring systems such as FIB-4 and baseline US or MRE-derived liver stiffness measurements may play a crucial role in identifying MASLD patients at risk of liver-related outcomes and death [29,30,53,54,55,56,57]. Data suggests that these non-invasive tests have a broader application beyond assessing liver fibrosis, as they can also be used to stratify patients at higher risk of hepatic decompensation. This information can be valuable in managing patients requiring closer clinical attention and in formulating cost-effective treatment paradigms. The MEFIB index, integrating FIB-4 and MRE, was initially developed to identify patients with significant fibrosis (≥stage 2) who would benefit from pharmacological therapy for MASLD [58]. In a study involving 1707 MASLD patients that had a median of 3 years of follow-up, Ajmera et al. found that a positive MEFIB index was associated with a hazard ratio of 20.5 (95% CI, 104 to 40.8, *p* < 0.01) for the development of the primary outcome as defined by ascites, hepatic encephalopathy or varices requiring treatment [29]. In contrast, the authors found a negative MEFIB index associated with a <1% risk of liver-related outcomes at 3 years. In our triple screen cohort, a positive MEFIB index was observed in 57 (27.5%) of 207 patients, suggesting that over a quarter are at high risk and may require closer clinical attention.

The triple screen MRI utilizes an abbreviated protocol involving a limited set of sequences. For morphologic assessment, the protocol only includes a 6 mm thick coronal T2 weighted sequence, a 10 mm thick axial T2 weighted sequence, and 3 mm thick axial chemical-shift in-phase and opposed phased T1 weighted Dixon sequences. Deliberately omitted from the protocol are a thin slice T2 weighted sequence, diffusion-weighted sequences, and post-gadolinium sequences to streamline the process and save time. Consequently, the triple screen MRI falls short of providing a standard diagnostic abdominal MRI assessment for focal lesion detection and characterization. Nevertheless, radiologists must exercise vigilance and identify potential incidentalomas that may necessitate a recommendation in the radiology report for follow-up with a complete MR study at a later date. Our study revealed incidental findings in 40 (14.9%) of the 268 MRI examinations, including 14 new findings and 30 known findings. Importantly, two cases required potential clinical intervention, including one new case of choledocholithiasis with upstream biliary obstruction and one new case of renal cell carcinoma.

The main study limitations include the study’s retrospective design and the lack of available clinical outcome data. Additionally, the generalizability of our findings may be limited to other tertiary care institutions, as our institution is a provincial referral center for CLD and hemochromatosis, which may skew our patient population. Nevertheless, this study provides data from a reasonably sized population on the liver triple screen MRI imaging innovation, reducing the knowledge gap in the literature regarding the experiences of large-volume clinical centers that perform comprehensive combined evaluations of liver fat, iron, and fibrosis in patients with CLD.

## 5. Conclusions

Our work has shown that the liver triple screen MRI examination is an effective method for comprehensively evaluating liver fat, iron, and fibrosis in patients with CLD. In clinical practice, it is common to encounter combined parenchymal disorders involving various combinations of fibrosis, fat, or iron in patients with CLD and, in particular, cohorts with MASLD. The liver triple screen technique, especially when using the SE-EPI MRI sequence, is technically robust and can be performed successfully in patients with a large body habitus and in challenging cases where US elastography has failed or been unreliable. This is of clinical relevance given the rising obesity epidemic globally. Implementing triple screen MRI can offer important clinical insights and is particularly valuable in identifying MASLD patients at risk for liver-related complications. For further research into triple screen MRI, it will be important to focus on the longitudinal monitoring of the disease process, integration of clinical outcome data into the analysis, and assessment in large multicenter studies.

## Figures and Tables

**Table 3:** Laboratory Data.

	Mean ± Standard Deviation	Normal
**Aspartate transaminase**	48.1 ± 42.6 U/L (range 11 to 324)	<45 U/L
**Alanine transaminase**	52.4 ± 52.1 U/L (range 8 to 403)	<70 U/L
**Albumin**	39.0 ± 4.6 g/L (range 15 to 49)	35 to 50 g/L
**Bilirubin**	16.8 ± 15.4 µmol/L (range 3 to 137)	<20 µmol/L
**Platelet**	212.0 ± 123.1 × 10^9^/L (range 41 to 1513 × 10^9^)	140–400 × 10^9^/L
**INR**	1.1 ± 0.3 (range 0.9 to 2.5)	0.8 to 1.2
**Sodium**	139.0 ± 10.2 mmol/L (range 4 to 147)	135 to 145 mmol/L
**Creatinine**	84.9 ± 25.4 µmol/L (range 39 to 229)	50 to 120 µmol/L
**Hemoglobin**	143.3 ± 18.3 g/L (range 59 to 182)	135 to 175 g/L
**Ferritin**	370.1 ± 489.1 µg/L (range 6 to 3147)	30 to 500 µg/L
**HbA1c**	6.4 ± 1.4% (range 4.2 to 12.4)	4.3 to 5.9%
**Triglyceride**	1.7 ± 0.9 mmol/L (range 0.5 to 5.2)	≤1.7 mmol/L
**Low-density lipoprotein**	2.2 ± 1.3 mmol/L (range 0.1 to 11.2)	≤3.4 mmol/L

**Table 4:** The Specialties of the Referring Physicians.

Referring Physician Groups	Percentage (%)
Gastroenterology	62.7
Family Medicine	17.2
Internal Medicine	14.6
Infectious Diseases	4.5
Pediatrics	0.7
General Surgery	0.4

## Data Availability

The original contributions presented in the study are included in the article, further inquiries can be directed to the corresponding author.
